# Long-term efficacy and safety of different corticosteroid courses plus mycophenolate mofetil for autoimmune encephalitis with neuronal surface antibodies without tumor

**DOI:** 10.3389/fimmu.2023.1195172

**Published:** 2023-07-10

**Authors:** Dong Li, Teng Huang, Fangyuan Zhang, Xiaoyu Zhang, Jingjing Dou, Chunjuan Wang, Shougang Guo

**Affiliations:** ^1^ Department of Neurology, Shandong Provincial Hospital, Shandong University, Jinan, Shandong, China; ^2^ Department of Neurology, Shandong Second Provincial General Hospital, Jinan, Shandong, China; ^3^ Department of Neurology, Shandong Provincial Hospital affiliated to Shandong First Medical University, Jinan, Shandong, China

**Keywords:** autoimmune encephalitis, neuronal surface antibodies, corticosteroids, mycophenolate mofetil, courses, efficacy and safety

## Abstract

**Objective:**

To compare the efficacy and safety of different-course corticosteroids plus mycophenolate mofetil (MMF) as maintenance therapy in autoimmune encephalitis (AE) with neuronal surface antibodies (NSAbs) without tumor and explore the optimal course of corticosteroids.

**Methods:**

Fifty-five patients with definite AE without tumor were enrolled consecutively between June 2015 and November 2020 and retrospectively divided three groups according to the course of treatment with corticosteroid, i.e., a group of patients with a course of 3-6 months (Group 3-6mo), 6-12 months (Group 6-12mo), and >12 months (Group >12mo). Demographic data, clinical manifestation and ancillary tests results were recorded. The dosage and courses of corticosteroid treatment, the recovery of neurological function, the occurrence of adverse effects, and relapses were followed up.

**Results:**

A total of 55 patients were included in the final analysis. The numbers of patients in Group 3-6 mo, Group 6-12 mo, and Group >12 mo was 14, 17, and 24, respectively. A significantly higher proportion of patients in Group >12 mo showed a decreased level of consciousness at the onset (12, 50%) than in Group 3-6 mo and Group 6-12 mo (2,14.3%; 3, 17.6%) (p = 0.033). The incidence of MRI abnormalities was significantly higher in Group 6-12 mo and Group >12 mo (10, 58.8%; 16, 66.7%) than in Group 3-6 mo (3, 21.4%) (P=0.023). Ordinal regression analysis indicated that decreased level of consciousness was associated with the course of corticosteroid (OR=3.838, 95% CI: 1.103-13.323, P=0.035). No significant difference was observed between the three groups regarding the cumulative dose of corticosteroids administered during the first three months of long-term treatment (P>0.05). Additionally, no significant difference in the cumulative dosage of corticosteroids was found between patients in Group 6-12 months and Group >12 months during the first 6 months after beginning long-term treatment. The mRS scores of the three groups were not statistically significant before and after first-line treatment or at the last follow-up. Bonferroni multiple comparison test indicated that the mRS scores of patients in Group 6-12 months and Group >12 months were not statistically significant at 3 months and 12 months after the start of long-term treatment. During the follow-up, 50 (90.9%) patients achieved satisfactory neurological function (mRS score ≤2). Five patients (9.1%) experienced a first relapse and 2 of them were overlapped with both anti-NMDA receptor and glial antibodies. The incidence of adverse effects was significantly higher in Group >12 mo (17, 70.8%) than in Group 3-6 mo (3, 21.4%) and Group 6-12 mo (5, 29.4%) (P=0.003).

**Conclusions:**

The beneficial effects of oral corticosteroid treatment may do not persist beyond 12 months and may even contribute to an increased incidence of adverse effects. In order to optimize the effectiveness and safety of treatment, we recommend a corticosteroid course of 3-12 months. Patients with reduced levels of consciousness may be more inclined to choose longer courses of corticosteroids for long-term treatment. Patients with an “overlapping syndrome” may require more intense immunotherapy to prevent relapse.

## 1 Introduction

Autoimmune encephalitis (AE) is a group of disorders in which the immune system attacks self-antigens expressed in the central nervous system (CNS) (1–3). Autoantibodies targeting nuclear and cytoplasmic proteins such as Hu, Ma, and Ri usually accompany malignancy. In contrast, neuronal surface antibodies (NSAbs) target synaptic receptors or components of synaptic protein complexes, including N−methyl−D−aspartate receptor (NMDAR), leucine-rich glioma inactivated-1 (LGI1), the cortactin-associated protein like 2 (CASPR2), α-amino-3-hydroxy-5-methyl-4-isoxazolepropionic acid receptor (AMPAR), gamma-aminobutyric acid (GABA)-A and -B receptors, dipeptidyl-peptidase-like protein-6 (DPPX), and glycine receptor (GlyR) (4, 5). Unlike encephalitis with antibodies to intracellular antigens, cases involving NSAbs have a relatively lower frequency of tumors (6). Patients with NSAbs generally respond well to immunotherapy and have a better overall prognosis (7). In the study, we use the term “AE” to refer only to AE with NSAbs and without tumors.

Common first-line immunotherapies for AE include corticosteroids, intravenous immunoglobulins (IVIG), and plasma exchange during the acute phase (2). To prevent early relapse, abrupt withdrawal of immunotherapy should be avoided after acute treatment (8–10). After the acute phase, sustained use of oral corticosteroids, azathioprine, and mycophenolate mofetil (MMF) may be used for sustained remission (9–11). Based on the Autoimmune Encephalitis Alliance Clinician Network survey results, rituximab emerges as the most frequently employed long-term immunosuppressive agent for autoimmune encephalitis (11). However, it is important to note that the use of rituximab in China for autoimmune encephalitis is considered off-label. Factors such as cost, hospitalization requirements, and concerns regarding potential side effects pose limitations to its widespread utilization in China (12). In contrast, MMF represents a more accessible long-term immunotherapy option for patients with autoimmune encephalitis. Corticosteroids can be used as a bridging strategy, followed by a gradual taper over weeks to months, or even years, overlapping with long-term immunotherapy after completing acute therapy. The combination of corticosteroids and MMF is a common long-term immunotherapy, with MMF needing to overlap with corticosteroids for at least 3-6 months due to its delayed onset of action (11, 13).

However, the efficacy and safety of long-term immunosuppression with oral agents remain to be evaluated. The appropriate duration of maintenance corticosteroid therapy is currently unknown, and the course of empirical use ranges widely from several months to several years, depending on the patient’s status and clinician’s opinion. Therefore, this study aims to compare the efficacy and safety of different-course corticosteroids plus MMF as maintenance therapy in AE without tumor and explore the optimal course of corticosteroids.

## 2 Methods

### 2.1 Patients and data collection

In this study, patients with definite AE were enrolled consecutively at Shandong Provincial Hospital in China between June 2015 and November 2020. The patients were retrospectively divided into three groups according to the course of treatment with corticosteroids, i.e., a group of patients with a course of 3-6 months (Group 3-6mo), 6-12 months (Group 6-12mo), and >12 months (Group >12mo). The inclusion criteria were as follows: 1) age≥ 16 years; 2) All patients included in the study were screened for autoimmune encephalitis associated antibodies in serum and cerebrospinal fluid (CSF) and had positive NSAbs in serum or CSF. All patients were diagnosed with neurosurface antibody-associated autoimmune encephalitis according to established diagnostic criteria (14); 3) maximal modified Rankin Scale (mRS) ≥ 3 at symptom onset; 4) first-line immunotherapy (steroids, IVIG, or combined) responded well; 5) maintenance immunotherapy with tapering corticosteroid and MMF (MMF continued for more than 12 months); 6) reasonable exclusion of other disorders. Exclusion criteria were defined: 1) patients who received non-first-line therapy during the acute phase or were treated with medications other than mycophenolate mofetil (MMF) and corticosteroids during the long-term immunotherapy phase; 2) patients with tumor; 3) lack of follow-up data; 4) patients who did well on immunotherapeutic treatments and discontinued treatments on their own.

In addition to tracking disease progression, we recorded the co-occurrence of various symptoms such as fever, headache, flu-like symptoms, or gastrointestinal issues. Demographic information and results of ancillary tests, including age at onset, sex, disease course, CSF tests, MRI, and EEG results, were also documented. We monitored the dosage and courses of corticosteroid treatment, the recovery of neurological function and relapses during the follow-up period. Additionally, we calculated the cumulative dose of prednisone for each group during different time periods. Adverse effects resulting from corticosteroid use were systematically collected and assessed throughout the study period. The targeted adverse effects included but were not limited to common symptoms such as weight gain, hyperglycemia, hypertension, osteoporosis and digestive symptoms. For our cohort, hypertension was defined as newly elevated blood pressure, re-elevation of blood pressure in the context of pre-existing hypertension, or when previously controlled hypertension required medication adjustments during immune maintenance therapy. Hyperglycemia was similarly defined. These effects were evaluated through clinical assessments, self-reporting by the patients, and accounts provided by their family members or caregivers (15, 16). In areas where clear recommendations were lacking, expert opinion was sought and consensus was reached among the investigators.

Furthermore, all patients underwent at least one screening for systemic tumors at onset and once per year during follow-up. The first-line immunotherapy options consisted of intravenous corticosteroid therapy (either 1000mg or 500mg of methylprednisolone for 3 or 5 days) and/or IVIG (0.4g/kg/day for each course for 5 days). Long-term therapy was defined as taking MMF at a dose of 1-2g daily for more than 12 months. Corticosteroid (prednisone) doses were tapered in combination with MMF and withdrawn once the maximum effect of the MMF became evident, based on clinical experience.

Clinical presentation incudes early onset (<45 years) and late onset (≥45 years) (17). Early treatment was defined as the initiation of immunotherapy within 30 days of onset. Treatment effects and long-term outcomes were assessed using mRS. The improvement of clinical manifestations was defined by an mRS score decrease of at least 1 (18). Favorable outcome was defined as an mRS score ≤2, and poor outcome was defined as an mRS score >2 at the last follow-up. Relapse was defined as an exacerbation of previous symptoms or the occurrence of new symptoms after being stable for 2 months (12). In patients with autoimmune encephalitis, the recurrence of epilepsy should be evaluated through comprehensive clinical assessment. For individuals presenting with acute symptomatic episodes of autoimmune encephalitis, the manifestation of disease recurrence is considered (19).

### 2.2 Statistical analysis

Statistical analyses were conducted using SPSS IBM 25.0, and GraphPad Prism 6.0 was used to generate figures. Quantitative data with normal distributions were presented as mean ± SD, while non-normally distributed data were presented as medians with the interquartile range (IQR). The Wilcoxon test was used to compare mRS scores before and after treatment. Symptoms, demographic data, and ancillary test results were analyzed using the χ2 test or Fisher exact test for categorical variables and Kruskal-Wallis H test for continuous variables. The bonferroni multiple comparison test after Kruskal-Wallis test was used for comparison between groups. One-way repeated measures ANOVA followed by Tukey multiple comparisons test for between group comparisons. Multinomial logistic regression analysis was used to assess factors affecting outcome. A p-value of less than 0.05 was considered significant.

## 3 Results

### 3.1 Clinical characteristics

A total of 55 patients were enrolled, all of whom met the 2016 diagnostic criteria for AE (14). Of these patients, 14 were in Group 3-6 mo, 17 were in Group 6-12 mo, and 24 were in Group >12 mo. The average age at onset of all patients was 48.21 ± 17.39 years and 65.5% were female while 34.5% were male. Early-onset (age <45 years) was present in 49.1% of patients, while late-onset (age ≥45 years) was present in 50.9%.

Prodromal symptoms, including fever, headache, flu-like symptoms, or gastrointestinal symptoms, were present in 19 patients (34.5%). Seizures were the most common clinical manifestation of AE, present in 42 patients (76.4%), followed by cognitive impairment (24 patients, 43.6%) and psychosis (23 patients, 41.8%). The proportion of patients with a decreased level of consciousness was significantly higher in Group >12 mo (12 patients, 50%) compared to the other groups (2 patients, 14.3%; 3 patients, 17.6%) (P=0.033). Further details on the clinical characteristics of the patients are provided in **Table 1**.

**Table 1:** Clinical characteristics of patients with AE.

	Total, n=55	3~6mo, n=14	6~12mo, n=17	>12 mo, n=24	P value
Age at onset, y, median	43.33 ± 17.25	37.14 ± 17.51	41.53 ± 15.74	48.21 ± 17.39	0.142
Sex					0.726
Male	36 (65.5%)	8 (57.1%)	12 (70.6%)	16 (66.7%)	
Clinical presentation					0.118
Early onset	27 (49.1%)	9 (64.3%)	10 (58.8%)	8 (33.3%)	
Late onset	28 (50.9%)	5 (35.7%)	7 (41.2%)	16 (66.7%)	
Prodromal symptoms^a^	19 (34.5%)	4 (28.6%)	7 (41.2%)	8 (33.3%)	0.820
Main symptoms					
Psychosis	23 (41.8%)	5 (35.7%)	6 (35.3%)	12 (50.0%)	0.557
Seizures	42 (76.4%)	9 (64.3%)	13 (76.5%)	20 (83.3%)	0.473
Cognitive impairment	24 (43.6%)	6 (42.9%)	7 (41.2%)	11 (45.8%)	1.000
Dysphasia	11 (20.0%)	4 (28.6%)	5 (29.4%)	2 (8.3%)	0.161
Dyskinesia	19 (34.5%)	6 (42.9%)	7 (41.2%)	6 (25.0%)	0.422
Decreased level of consciousness	17 (30.9%)	2 (14.3%)	3 (17.6%)	12 (50%)	0.033*
Automomic dysfunction	6 (10.9%)	1 (7.1%)	3 (17.6%)	2 (8.3%)	0.641

a Fever, headache, flu-like symptoms, or gastrointestinal symptoms (vomiting/diarrhea). *p < 0.05. Clinical presentation incudes early onset (<45 years) and late onset (≥45 years).

### 3.2 Ancillary test results

All 55 patients (100%) were tested for autoantibodies to NSAbs with paired CSF and serum, and the following antibody findings were present at diagnosis of AE: NMDAR in 26 patients (47.3%), LGI1 in 23 patients (41.8%), GABA_B_R in 4 patients (7.3%), CASPR2 in 1 patient (1.8%), and mGluR5 in 1 patient (1.8%). One NMDAR-AE patient was positive for aquaporin-4 (AQP4) antibody and another NMDAR-AE patient was positive for myelin oligodendrocyte glycoprotein (MOG) antibody (**Table 2**).

**Table 2:** Summary of auxiliary examination results.

	Total, n=55	3~6mo, n=14	6~12mo, n=17	>12 mo, n=24	P value
Classified by antibodies					0.567
NMDAR	26 (47.3%)	8 (57.1%)	8 (47.1%)	10 (41.7%)	
LGI1	23 (41.8%)	4 (28.6%)	8 (47.1%)	11 (45.8%)	
GABA_B_R	4 (7.3%)	2 (14.3%)	0 (0.0%)	2 (8.3%)	
CASPR2	1 (1.8%)	0 (0.0%)	0 (0.0%)	1 (4.2%)	
mGluR5	1 (1.8%)	0 (0.0%)	1 (5.9%)	0 (0.0%)	
Brain MRI abnormalities at diagnosis	29 (52.7%)	3 (21.4%)	10 (58.8%)	16 (66.7%)	0.023*
Medial temporal lobe	22 (40.0%)	1 (7.1%)	7 (41.2%)	14 (58.3%)	0.007*
Frontal lobe	7 (12.7%)	1 (7.1%)	2 (11.8%)	4 (16.7%)	0.877
Parietal lobe	6 (10.9%)	2 (14.3%)	1 (5.9%)	3 (12.5%)	0.749
Occipital lobe	1 (1.8%)	0 (0.0%)	0 (0.0%)	1 (4.2%)	1.000
Insula	6 (10.9%)	1 (7.1%)	2 (11.8%)	3 (12.5%)	1.000
Diencephalon	1 (1.8%)	0 (0.0%)	1 (5.9%)	0 (0.0%)	0.564
Basal ganglia	6 (10.9%)	1 (7.1%)	4 (23.5%)	1 (4.2%)	0.182
Cerebellum	1 (1.8%)	0 (0.0%)	0 (0.0%)	1 (4.2%)	1.000
Brainstem	2 (3.6%)	0 (0.0%)	1 (5.9%)	1 (4.2%)	1.000
Demyelination	5 (9.1%)	1 (7.1%)	2 (11.8%)	2 (8.3%)	1.000
EEG^b^	39 (70.9%)	11 (78.6%)	11 (64.7%)	17 (70.9%)	0.646
Epileptic discharges	7 (13.5%)	2 (14.3%)	4 (25.0%)	1 (4.5%)	0.202
Slow activity	21 (40.4%)	4 (28.6%)	6(37.5%)	11 (55.0%)	0.422
Delta-brush	5 (9.6%)	1 (7.1%)	1 (6.3%)	3 (13.6%)	0.851
CSF					
WBC in CSF (*10^6^)	5 (2-25)	6 (3-26)	7 (1-20.5)	5 (2.25-21.25)	0.820
Pleocytosis (mononuclear cells>5*10^6^)	27 (49.1%)	7 (50.0%)	9 (52.9%)	11 (45.8%)	0.941
Protein	0.32 (0.24-0.47)	0.285 (0.19-0.41)	0.32 (0.235-0.435)	0.35 (0.28-0.69)	0.233
Increased level of protein(>0.45g/L)	14 (25.5%)	3 (21.4%)	4 (23.5%)	7 (29.2%)	0.857

b Video-electroencephalogram (V-EEG) was performed in 52 patients. *p < 0.05. NMDAR, N-methyl-D-aspartate receptor; LGI1, leucine-rich glioma-inactivated protein 1; GABA_B_R, g-aminobutyric acid receptor B; CASPR2, contactin-associated protein-like 2; mGluR5, metabotropic glutamate receptor 5; CSF, cerebrospinal fluid; WBC, white blood cell.

All patients underwent brain MRI at onset, with 29 patients (52.7%) showing abnormal fluid-attenuated inversion recovery sequences. The medial temporal lobe was most prone to abnormalities, observed in 22 patients (40.0%). The proportion of patients with abnormal MRI of the medial temporal lobe was lower in Group 3-6 mo (1 patient, 7.1%) compared to Group 6-12 and Group >12 mo (7 patients, 41.2%; 14 patients, 58.3%) (P=0.007). Other involved areas included the frontal, parietal, and occipital lobes, insula, diencephalon, cerebellum, and brainstem. Demyelinating lesions were observed in 5 patients (9.1%) with or without associated demyelinating antibodies (**Table 2**).

EEG was performed in all patients, with video-EEG performed in 52 patients. Abnormal EEG findings were seen in 39 patients (70.9%), with slow activity present in 21 patients (40.4%), epileptic discharges in 7 patients (13.5%), and delta-brush in 5 patients (9.6%), with 4 of these patients having anti-NMDAR encephalitis (**Table 2**).

Lumbar punctures were performed in all patients for diagnosis, with CSF results at onset collected and analyzed before immunotherapy. An analysis of the results for 55 patients showed that 27 (49.1%) having pleocytosis and a median white blood cell count of 5 (IQR 2-25). The median CSF protein content was 0.32 (IQR 0.24-0.47) g/L, with 14 patients having an increased protein level (**Table 2**).

### 3.3 General hospitalization status and treatment outcomes

**Figure 1:**
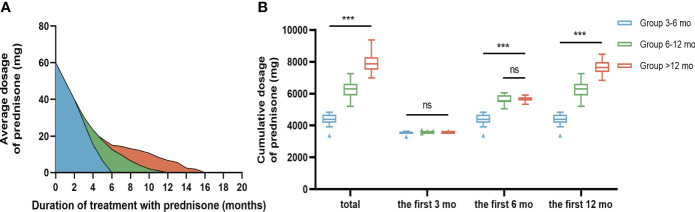
Comparison of oral prednisone dosage in three groups. **(A)** Changes of average oral prednisone dose in different time periods. **(B)** Comparison of cumulative prednisone dosage in three groups. During the long-term treatment phase, the total dose of bonisone acetate differed significantly among the three groups in descending order: Group >12 months, Group 6-12 months, and Group 3-6 months (P<0.001). During the first three months of long-term treatment, the cumulative oral prednisone dose was not statistically significant between the 3 groups **(B)**. During the first six months of long-term treatment, the cumulative dose of oral prednisone between Group 6-12 mo and Group >12 mo was not statistically significant **(B)**. Data is visualized as Tukey box plots (line at mean, top of the box at the 75th percentile, bottom of the box at the 25th percentile, whiskers at the highest and lowest values, outliers shown as triangles beyond the whiskers). "***p < 0.001, ns P>0.05. One-way repeated measures ANOVA followed by Tukey multiple comparisons test for between group comparisons.

The median duration from onset to initiation of immunotherapy was 30 days (IQR 15-77). The mean duration of hospitalization was 16.55±7.047 days. Of the total patients, 35 (63.6%) received a combined regimen of corticosteroid pulse and IVIG, while 20 (36.4%) only received corticosteroid pulse (methylprednisolone). None of the patients treated with plasma exchange in our study met the inclusion criteria. During the long-term treatment phase, there was a significant difference in the total dosage of prednisone acetate among the three groups, with the order being Group >12 months, Group 6-12 months, and Group 3-6 months (P<0.05, **Figure 1**). Pairwise comparisons between the three groups also yielded statistically significant results (P<0.05, **Figure 1**). Conversely, no significant difference was observed between the three groups regarding the cumulative dose of corticosteroids administered during the first three months of long-term treatment (P>0.05, **Figure 1**). Additionally, no significant difference in the cumulative dosage of corticosteroids was found between patients in Group 6-12 months and Group >12 months during the first 6 months after beginning long-term treatment (P>0.05, **Figure 1**) (**Table 3**).

**Table 3:** General hospitalization status and follow-up of patients.

	Total, n=55	3~6mo, n=14	6~12mo, n=17	>12 mo, n=24	P value
Duration from onset to initiation of immunotherapy	30.00 (15.00-77.00)	26.50(17-46.25)	29.00 (11.5-102.50)	35.50 (18.50-90.00)	0.452
Early treatment	28 (50.9%)	9 (64.3%)	10 (58.8%)	9 (37.5%)	0.428
Length of hospitalization	16.55 ± 7.047	18.64 ± 5.429	15.53 ± 6.453	16.04 ± 8.201	0.432
Follow-up time	35.47 ± 7.714	34.64 ± 7.682	36.29 ± 7.671	35.38 ± 8.032	0.841
Acute immunotherapy					0.590
corticosteroids	20 (36.4%)	4 (28.6%)	8 (47.1%)	8 (33.3%)	
IVIG+corticosteroids	35 (63.6%)	10 (71.4%)	9 (52.9%)	16 (66.7%)	
Dosage of prednisone (mg)					
Dosage of the first 3 mo	3567.45 ± 52.11	3540.00 ± 85.58	3582.35 ± 44.52	3572.92 ± 14.29	0.115
Dosage of the first 6 mo	5326.91 ± 633.97	4350 ± 403.39	5633.53 ± 278.21^#^	5679.58 ± 132.75^#^	0.000*
Dosage of the first 12 mo	6376.91 ± 1404.51	4350 ± 403.39	6268.82 ± 538.46	7635.83 ± 417.81	0.000*
Total dosage	6499.64 ± 1539.98	4350 ± 403.39	6268.82 ± 538.46	7917.08 ± 570.67	0.000*
mRS					
maximal mRS at admission (IQR)	4 (4-5)	4 (4-5)	4 (4-5)	5 (4-5)	0.540
mRS after first-line treatment (IQR)	3 (2-3)	2 (2-3)	3 (2-3)	3 (2-3)	0.156
mRS at 3 mo (IQR)	2 (2-2)	1 (1-1.25)	2 (2-2) ^#^	2 (2-2) ^#^	0.000*
mRS at 6 mo (IQR)	1 (1-2)	0 (0-1)^#^	1 (1-1)^#^	2 (2-2)	0.000*
mRS at 12 mo (IQR)	1 (0-1)	0 (0-1)^†^	0 (0-1) ^†,#^	1 (1-1) ^#^	0.023*
mRS score at final follow-up (IQR)	1 (0-1)	0 (0-1)	0 (0-1)	0 (0-1)	0.134
mRS score ≤2 at final follow-up (n, %)	50 (90.9%)	13 (92.9%)	16 (94.1%)	22 (91.7%)	0.684
ΔmRS	4 (3-4)	4 (3-4)	4 (3-4)	4 (3-4)	0.156
Relapse (n, %)	5 (9.1%)	1 (7.1%)	1 (5.9%)	3 (12.5%)	0.849

^†^,^#^ Both data were not statistically significant; *P<0.05.. IVIG, intravenous immunoglobulins; mRS, modified Rankin scale; ΔmRS, Changes in mRS from maximal onset to last follow-up; IQR, interquartile range.

**Figure 2:**
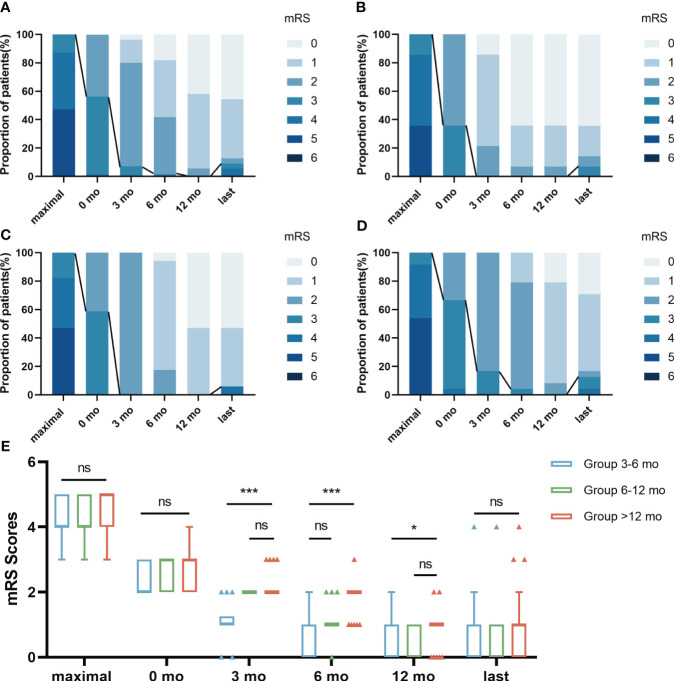
The changes of mRS scores in 3 groups during follow-up. The distribution of evaluation was depicted at 6 time points: “maximal” indicates the patient’s highest mRS Scores at the time of onset; 0 mo indicated a time point after first-line treatment; “3 mo” indicates the mRS Scores after 3 months of treatment with prednisone combined with MMF; “6 mo” indicates the mRS Score after 6 months of treatment with prednisone combined with MMF; “12 mo” indicates the mRS Score after 12 months of treatment with prednisone combined with MMF; “last” indicates the mRS Scores at the patient’s last follow-up. The line represented the change in scores dividing “favorable clinical outcome” of mRS scores ≤ 2. The results were applied to compare the distribution of mRS scores in total patients **(A)**, Group 3-6 mo **(B)**, Group 6-12 mo **(C)** and Group >12 mo **(D)**. The mRS Scores of the three groups were not statistically significant before and after first-line treatment or at the last follow-up **(E)**. Bonferroni multiple comparison test showed that mRS Scores of patients in Group 6-12 mo and Group >12 mo had no statistical significance at 3 months and 12 months after the start of long-term treatment **(E)**. The mRS Scores of patients in Group 3-6 mo and Group 6-12 mo were not statistically significant 6 months after the start of treatment **(E)**. Data is visualized as Tukey box plots (line at median, top of the box at the 75th percentile, bottom of the box at the 25th percentile, whiskers at the highest and lowest values, outliers shown as triangles beyond the whiskers). *p < 0.05, ***p < 0.001, ns P>0.05. The bonferroni multiple comparison test after Kruskal-Wallis test was used for comparison between groups.

The median maximal mRS at symptom onset was 4 (IQR 4-5), significantly higher than the score of 1 (IQR 0-1) at best recovery state and final follow-up (P< 0.05). During a mean follow-up of 35.47 ± 7.71 months, 50 (90.9%) patients achieved satisfactory neurological function (mRS score ≤2) (**Figure 2**). The mRS scores of the three groups were not statistically significant before and after first-line treatment or at the last follow-up (P>0.05, **Figure 2**). The Kruskal-Wallis test showed statistically significant differences in mRS scores between the three groups at 3 months, 6 months, and 12 months after the start of long-term treatment (P<0.05, **Figure 2**). Bonferroni multiple comparison test indicated that the mRS scores of patients in Group 6-12 months and Group >12 months were not statistically significant at 3 months and 12 months after the start of long-term treatment (P>0.05, **Figure 2**). The mRS scores of patients in Group 3-6 months and Group 6-12 months were not statistically significant 6 months after the start of treatment (P>0.05, **Figure 2**). For more detailed information, please refer to **Table 3**.

### 3.4 Factors associated with corticosteroid courses

To identify factors associated with different courses of patients with AE, we performed an ordinal regression analysis on data from the three groups. Univariate analysis indicated that decreased level of consciousness (P=0.033), brain MRI abnormalities (P=0.023), and MRI abnormalities in the temporal lobe (P=0.007) were associated with significant differences in corticosteroid course. These factors were included in the ordered logistic regression model, and the results showed that decreased level of consciousness was associated with corticosteroid courses (odds ratio (OR)=3.838, 95% confidence interval (CI): 1.103-13.323, P=0.035).

### 3.5 Relapse

**Figure 3:**
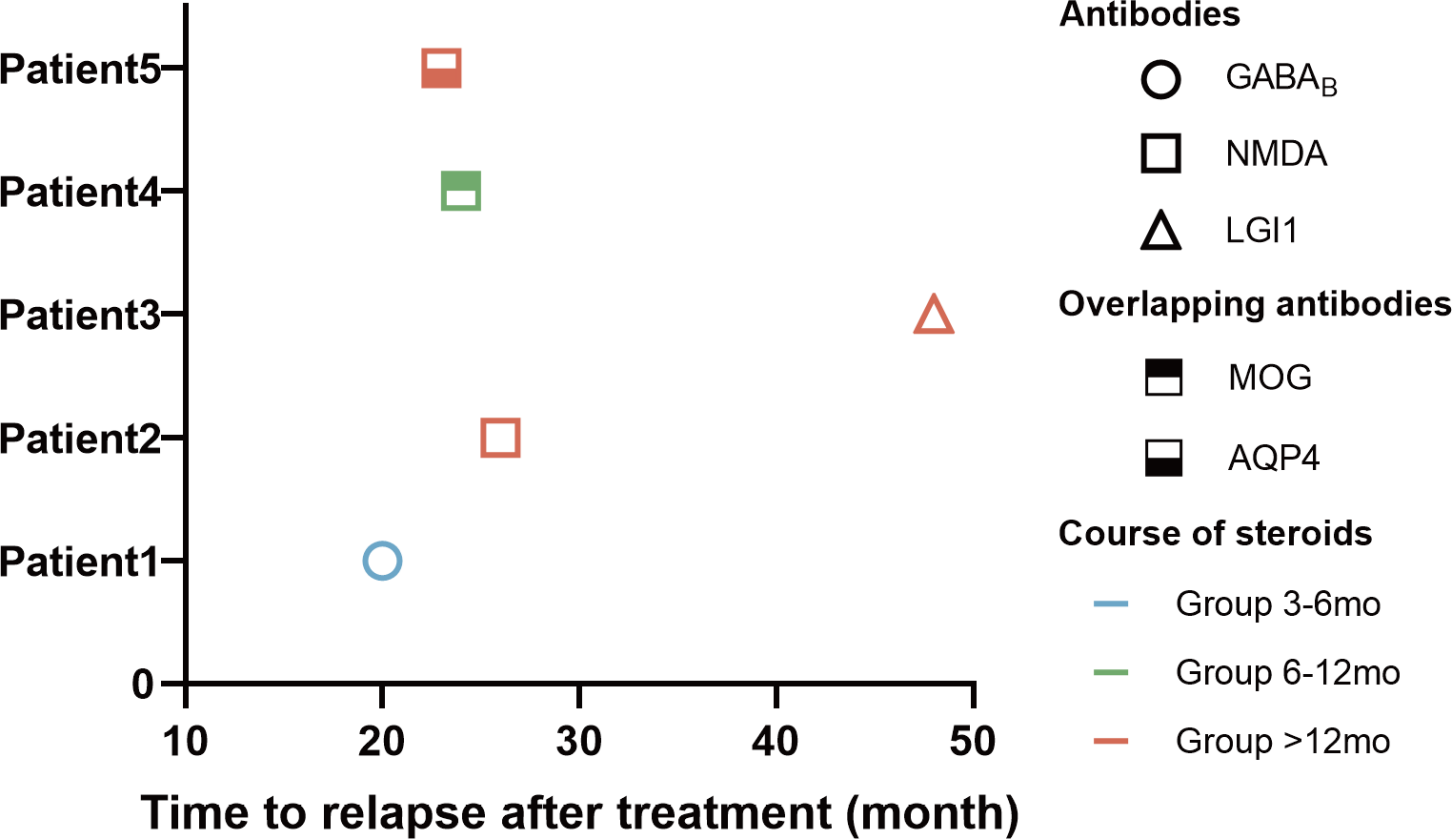
Clinical data of 5 cases with relapse of AE. The horizontal axis of the graph represents the time to relapse for patients who underwent immunotherapy. The data is categorized based on the type of antibodies, the duration of hormone use, and the time elapsed until disease relapse.

During a mean follow-up of 35.47 ± 7.71 months, five patients (9.1%) experienced a first relapse (**Table 3**). The number of people who experienced relapse was 1 (7.1%), 1 (5.9%), and 3 (12.5%) in Group 3-6 mo, Group 6-12 mo, and Group >12 mo, respectively (P>0.05). Of the relapsed patients, three (60%) were male and two (40%) were female. The most common symptoms among the relapsed patients were a decreased level of consciousness (5/5, 100%), cognitive impairment (4/5, 80%), and seizures (3/5, 60%). The three relapsing patients who developed epilepsy had another acute symptomatic seizures when their disease recurred. Three (3/5, 60%) of the relapsed patients were positive for anti-NMDAR antibodies, and 2 of them had both anti-NMDA receptor (NMDAR) and glial antibodies (**Figure 3**). One (1/5, 20%) relapsed patient was positive for anti-GABA_B_R antibody and the other (1/5, 20%) was positive for anti-LGI1.The median duration from the first-line treatment to the first relapse was 23 months (range, 20-48 months). The majority of patients experienced their first relapse within the initial 24 months (3/5, 60.0%).

### 3.6 Adverse effects

Adverse effects were reported in 25 (45.5%) patients, with the majority of cases (15/55, 27.3%) being weight gain. Other adverse effects included hyperglycemia (6/55, 10.9%), osteoporosis (4/55, 7.3%), hypertension (3/55, 5.5%), digestive symptoms (2/55, 3.6%), corticosteroid-related necrosis of the femoral head (2/55, 3.6%), abnormal liver function (2/55, 3.6%), and opportunistic infection (1/55, 1.8%). The incidence of adverse effects in the three groups was 21.4%, 29.4%, and 70.8%, respectively (P=0.003). A two-by-two comparison of the three groups showed a statistically significant difference in the proportion of patients who experienced adverse effects in Group >12 mo compared to Group 3-6 mo (P=0.004) and Group 6-12 mo (P=0.012) (**Table 4**).

**Table 4:** Patient adverse effects summary for corticosteroid and MMF therapy.

	Total, n=55	3~6mo, n=14	6~12mo, n=17	>12 mo, n=24	P value
Adverse effects	25 (45.5%)	3 (21.4%)	5 (29.4%)	17 (70.8%)	0.003*
Weight gain	15 (27.3%)	1 (7.1%)	3 (17.6%)	11 (45.8%)	0.029*
Hyperglycemia	6 (10.9%%)	1 (7.1%)	3 (17.6%)	2 (8.3%)	0.641
Osteoporosis	4 (7.3%)	0 (0.0%)	1 (5.9%)	3 (12.5%)	0.454
Hypertension	3 (5.5%)	0 (0.0%)	2 (11.8%)	1 (4.2%)	0.456
Digestive symptoms	2 (3.6%)	1 (7.1%)	0 (0.0%)	1 (4.2%)	0.725
Corticosteroid-related necrosis of the femoral head	2 (3.6%)	1 (7.1%)	0 (0.0%)	1 (4.2%)	0.251
Abnormal liver function	2 (3.6%)	0 (0.0%)	1 (5.9%)	1 (4.2%)	1.000
Opportunistic infection	1 (1.8%)	0 (0.0%)	1 (5.9%)	0 (0.0%)	0.564

*p < 0.05.

## 4 Discussion

In this study, we describe the clinical features, ancillary findings, prognostic outcomes and adverse effects caused by corticosteroids in AE patients using different courses of corticosteroids. We found that decreased level of consciousness at the time of onset may associate with a prolonged course of corticosteroids. Patients treated with corticosteroids for more than 6 months had a higher rate of MRI abnormalities. The beneficial effects of oral corticosteroid treatment may do not persist beyond 12 months and may even contribute to an increased incidence of adverse effects.

The most common clinical manifestations of AE were seizures, cognitive impairment, decreased level of consciousness and psychosis in our cohort, and the most common antibody types in AE patients were anti-NMDAR and LGI1, which are consistent with previous studies [15-18]. In our cohort, a total of 17 cases (30.9%) experienced a decreased level of consciousness. The prevalence of decreased level of consciousness in patients with AE ranges from 23% to 53.2% among various cohorts (12, 20, 21). Reasons for heterogeneity may be explained by inclusion criteria, sample size, or other factors, including genetic background and epidemiology. Ordinal regression analysis showed that decreased level of consciousness was associated with corticosteroid courses. The results suggest that AE patients on long-term immunotherapy with corticosteroids combined with MMF may require a longer course of corticosteroids if they have decreased level of consciousness at the onset of the disease. The reason for this may be related to the fact that a decreased level of consciousness is often considered to be associated with a poor prognosis (12, 20, 21). In general, a diminished level of consciousness at the onset of AE may suggest more severe and diffuse inflammation in the brain, necessitating a more aggressive course of corticosteroid therapy. We also observed that patients with longer courses of treatment tended to have a later age of onset, although this difference did not reach statistical significance. Previous studies have suggested that disease severity may be related to age at onset, and that patients over 45 years of age may be at increased risk of death (22–24). Further research in larger clinical cohorts may be necessary to fully understand the relationship between age and the course of corticosteroid use in patients with AE.

Twenty-nine patients (52.7%) had abnormal fluid-attenuated inversion recovery sequence. MRI changes were mainly abnormal in the medial temporal lobe, which is consistent with previous studies in China (25). The incidences of MRI abnormalities in the three groups were 21.4%, 58.8% and 66.7%, respectively. As our data showed, patients treated with steroids for more than 6 months had a significantly higher incidence of MRI abnormalities at presentation. However, no significant relationship between MRI abnormalities and corticosteroid regimen was found in the regression analysis. The abnormalities in the MRI of the brain are further evidence that AE can involve the entire brain or only a few regions. The relationship between brain MRI abnormalities and prognosis in AE patients is unclear. Previous studies have showed that normal MRI presentation associated with remission of epilepsy at 6 months (26). The development of brain MRI changes might indicate a poor prognosis (27–29). To date, there have been no studies on the correlation between MRI changes and the course of corticosteroid use in patients with AE.

By analyzing the cumulative dose of corticosteroids administered during the long-term treatment phase, we observed that the cumulative dose of glucocorticoids within the initial 3 months did not exhibit a significant difference among the three groups. Furthermore, no significant distinction was found between Group 6-12 mo and Group >12 mo within the first 6 months. These findings effectively mitigate concerns related to potential confounding factors arising from variations in drug dosage during the corresponding follow-up periods (**Table 3**; **Figure 1**).

Most patients (90.9%) in our study had a long-term favorable outcome during the follow-up period (mRS ≤ 2). There was no significant difference in the favorable outcome and relapses of AE treated with different courses of corticosteroids. The mRS scores of the three groups were not statistically significant before and after first-line treatment or at the last follow-up. This suggests that the effect of first-line treatment in the acute phase and the final prognosis of long-course treatment are essentially the same for patients using different courses of corticosteroids. Bonferroni multiple comparison test indicated that the mRS scores of patients in Group 6-12 mo and Group >12 mo was not statistically significant at 3 months and 12 months after the start of long-term treatment (**Table 3**; **Figure 2**).

This suggests that the beneficial effects of oral corticosteroid treatment did not persist beyond one year. During the follow-up period, 5 (9.1%) patients experienced a relapse, 3 with anti-NMDAR encephalitis, 1 with anti-LGI1 encephalitis and 1 with anti-GABA_B_R encephalitis (**Figure 3**). All patients who relapse had impaired consciousness. Patients with anti-NMDAR encephalitis appeared to be more likely to relapse than patients with anti-LGI1 (25, 30). Most patients with anti-GABA_B_R encephalitis without tumors achieve self-care after immunotherapy. Elderly patients who have severe clinical symptoms or a poor prognosis may require extra attention for tumor screening (31). “Over-lapping syndrome” was identified in the two relapsed patients (3.6%) and both of them showed demyelination on MRI (1 AQ4 positive, 1 MOG positive). A small proportion of patients with anti-NMDAR encephalitis have a concurrent immune response against glial cell antigens that affects the prognosis of such patients (32).These patients may require more intense immunotherapy and early use of second-line immunotherapy (33, 34).

Long-term use of corticosteroids may result in a variety of side effects, such as weight gain, hypertension, hyperglycemia, opportunistic infections, and others. Thus, the efficacy and safety of corticosteroid therapy should be thoroughly assessed on a case-by-case basis, and patients should be closely monitored for any adverse reactions during the entire course of treatment. In our study, all patients received long-term immunotherapy consisting of a combination of corticosteroids and MMF. The dosing of corticosteroids was adjusted based on the individual patient’s clinical presentation, risk of relapse, treatment response, and tolerance, and was gradually tapered to reach stable levels or discontinued once MMF achieved its maximum therapeutic effect. During the course of long-term immunotherapy, 25 patients (45.5%) experienced adverse effects. Weight gain was the most frequently reported adverse effect among patients undergoing long-term immunotherapy with corticosteroids plus MMF, with additional adverse effects including digestive symptoms, hypertension, hyperglycemia, osteoporosis, corticosteroid-related necrosis of the femoral head, abnormal liver function, and opportunistic infection. The relatively low doses of oral corticosteroids received over an extended period of time by patients with AE receiving corticosteroid may contribute to the low incidence of opportunistic infections by helping to maintain a more robust immune system. Patients on corticosteroid courses lasting 3-6 months and 6-12 months experienced significantly fewer adverse effects compared to those on corticosteroids for more than 12 months. This suggests that the beneficial effects of oral corticosteroid treatment did not persist beyond one year and may even contribute to an increased incidence of adverse reactions. Therefore, our study suggests that in the course of long-term immunotherapy with corticosteroid drugs combined with MMF, the optimal course of corticosteroids in AE patients should be 3-12 months.

Although our study provides valuable insights into the outcomes of patients with AE, there are several limitations to our findings. Firstly, the sample size in our cohort was relatively small, which may have resulted in limited statistical power and reduced the generalizability of our findings. Additionally, while we used the mRS score as a measure of functional outcomes, it is important to note that this score may not fully capture the cognitive or behavioral changes that can occur in patients with AE. Additional parameters, such as CASE or other means of evaluating cognitive or behavioral function, may be necessary to provide a more comprehensive assessment of the outcomes of AE. Based on these observations, it is possible that the three groups under investigation may not be fully comparable in terms of several variables that could potentially act as confounding factors. This discrepancy in comparability needs to be taken into consideration when interpreting the study results and drawing conclusions. Overall, it is important to acknowledge these limitations when interpreting our findings, and future studies with larger sample sizes and more comprehensive outcome measures are needed to confirm and extend our results.

In summary, the beneficial effects of oral corticosteroid treatment did not persist beyond one year and may even contribute to an increased incidence of adverse reactions. In order to optimize the effectiveness and safety of treatment, we recommend a corticosteroid course of 3-12 months. It is important to note that decreased level of consciousness may increase the risk of requiring a prolonged course of corticosteroids. Patients with an “overlapping syndrome” at onset may require more intense immunotherapy to prevent relapse.

## Data availability statement

The raw data supporting the conclusions of this article will be made available by the authors, without undue reservation.

## Ethics statement

The studies involving human participants were reviewed and approved by the ethics institutional review board of Shandong Provincial Hospital. Written informed consent to participate in this study was provided by the participants’ legal guardian/next of kin. Written informed consent was obtained from the individual(s), and minor(s)’ legal guardian/next of kin, for the publication of any potentially identifiable images or data included in this article.

## Author contributions

DL, TH, CW and SG contributed to the study conception and design. Material preparation, data collection, and analysis were performed by DL, TH, FZ, XZ and JD. The first draft of the manuscript was written by DL, and all authors commented on previous versions of the manuscript. All authors contributed to the article and approved the submitted version.
